# Sub‐lineages of the SARS‐CoV‐2 Omicron variants: Characteristics and prevention

**DOI:** 10.1002/mco2.172

**Published:** 2022-08-16

**Authors:** Ailan Xu, Bixia Hong, Fuxing Lou, Shuqi Wang, Wenye Li, Amna Shafqat, Xiaoping An, Yunwei Zhao, Lihua Song, Yigang Tong, Huahao Fan

**Affiliations:** ^1^ College of Life Science and Technology Beijing University of Chemical Technology Beijing China; ^2^ The First Affiliated Hospital of Jiamusi University Jiamusi China

**Keywords:** immune escape, neutralizing antibodies, Omicron variant, SARS‐CoV‐2, vaccination

## Abstract

Since the start of the coronavirus disease 2019 (COVID‐19) pandemic, new variants of severe acute respiratory syndrome coronavirus 2 (SARS‑CoV‑2) have emerged, accelerating the spread of the virus. Omicron was defined by the World Health Organization in November 2021 as the fifth “variant of concern” after Alpha, Beta, Gamma, and Delta. In recent months, Omicron has become the main epidemic strain. Studies have shown that Omicron carries more mutations than Alpha, Beta, Gamma, Delta, and wild‐type, facilitating immune escape and accelerating its transmission. This review focuses on the Omicron variant's origin, transmission, main biological features, subvariants, mutations, immune escape, vaccination, and detection methods. We also discuss the appropriate preventive and therapeutic measures that should be taken to address the new challenges posed by the Omicron variant. This review is valuable to guide the surveillance, prevention, and development of vaccines and other therapies for Omicron variants. It is desirable to develop a more efficient vaccine against the Omicron variant and take more effective measures to constrain the spread of the epidemic and promote public health.

## Graphical abstract

In this article, we described the discovery of the Omicron variant, biological characteristics of the Omicron variant, immune escape, mutations, patients’ clinical features, vaccination, detection methods, treatment with drugs and neutralizing antibodies, and protective measures taken. It is desirable to develop a more efficient vaccine against the Omicron variant and take more effective measures to contain the spread of the epidemic and promote public health.

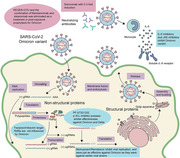

## 1 INTRODUCTION

Since December 2019, severe acute respiratory syndrome coronavirus 2 (SARS‐CoV‐2) has swept the world in various forms, with different mutations. As of July 22, 2022, there were 565,207,160 cases of coronavirus disease 2019 (COVID‐19) and 6,373,739 deaths (https://covid19.who.int/ [July 22, 2022]). Under the wall of vaccines, antibodies, and drugs, variants continue to arise, including five variants of concern (VOCs) (Alpha, Beta, Gamma, Delta, and Omicron) with increased transmissibility, virulence, or reduced diagnostic, therapeutic, and vaccine potency, which pose a potential threat to public health.

The Delta wave, which occupied a dominant position in most countries worldwide, gradually faded in South Africa and was replaced with the looming of a new variant—Omicron, and it has become the fourth peak driving the epidemic in South Africa (first peak: Alpha; second peak: Beta; third peak: Delta). Omicron was a newly emerged VOC, which possessed seven subvariants, including BA.1, BA.1.1, BA.2, BA.3, BA.2.12.1, BA.4, and BA.5. Currently, Omicron BA.2 is the main variant causing SARS‐CoV‐2 infections worldwide. However, the SARS‐CoV‐2 Omicron BA.2.12.1, BA.4, and BA.5 subvariants are phylogenetically independent of the BA.2 evolutionary branch. The Omicron BA.4 and BA.5 subvariants are currently at low endemic levels globally. The rapid spread of new subvariants of Omicron has led to a rapid increase in prevalence in the USA and South Africa, implying its potential for further global pandemics. The new subvariants of Omicron have unique mutational sites, which facilitate accelerating virus transmission. BA.4 and BA.5 were found in South Africa in December 2021 and January 2022, respectively.^1^ L452Q and S704L mutation sites were found in the spike protein of Omicron BA.2.12.1. Del69‐70, L452R, F486V, and R493Q mutation sites were found in the spike protein of BA.4 and BA.5, which accelerated the spread of the virus and enhanced pathogenicity.^1^ Current BA.4/BA.5 is replacing BA.2.12.1. Relevant studies have shown that BA.4 and BA.5 Omicron subvariants propagate faster compared with the other variants.^2^ A recent study published in *Science* found that people infected with Omicron produce insufficient titers of neutralizing antibodies against Omicron itself, and thus the idea of “Omicron infection as a natural vaccine” is unrealistic.^3^

The emergence of Omicron, especially the new subvariants, may reduce the effectiveness of current drugs and COVID‐19 vaccines. Omicron variants have attracted much attention worldwide and pose a serious threat to public health. This review aims to introduce Omicron variants in a more comprehensive, detailed, and timely manner, the characteristics of Omicron, mutation sites of known subvariants, and new subvariants. Meanwhile, the protective efficacy of existing drugs, antibodies, and COVID‐19 vaccines to Omicron will be summarized and may help to block the Omicron transmission and provide relevant theories and countermeasures for COVID‐19 therapy and ending epidemics.

## 2 CHARACTERISTICS OF THE OMICRON VARIANT

### 2.1 The outbreak and transmission of Omicron

**FIGURE 1:**
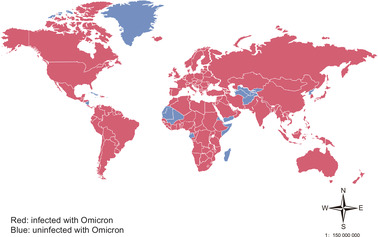
Main areas ravaged by Omicron. As of July 22, 2022, the Omicron variant has been detected in at least 187 countries and 50 US states. (https://outbreak.info/situation‐reports/omicron?loc = ZAF&loc = GBR&loc = USA&selected = Worldwide&overlay = false [July 22, 2022])

Omicron (B.1.1.529) was initially sequenced in South Africa. It was first reported by the World Health Organization (WHO) on the November 24, 2021, and then designated as a VOC by WHO on November 26, 2021 (https://www.who.int/news/item/26‐11‐2021‐classification‐of‐omicron‐(b.1.1.529)‐sars‐cov‐2‐variant‐of‐concern [November 26, 2021]). Until July 16, 2022, Omicron spreads to at least 185 countries all over the world (Figure 1). Seven subvariants of Omicron (BA.1, BA.1.1, BA.2, BA.3, BA.2.12.1, BA.4, and BA.5) with unique mutations have been identified. The first lineage officially designated as Omicron VOC is BA.1. The case of BA.2 with no 69/70 deletion in the spike protein region is growing in several countries such as India, South Africa, Denmark, and the United Kingdom. BA.1.1 carries an additional R346K mutation that is absent in other Omicron lineages, spike protein of the BA.3 contains fewer mutations, and current sequence monitoring data on BA.3 are limited.

It should be noted that BA.2 tended to outperform BA.1. Israeli news indicated that BA.2 was approximately 30% more infectious than BA.1 (https://www.haaretz.com/israel‐news/more‐infectious‐variant‐of‐omicron‐likely‐to‐become‐israel‐s‐dominant‐covid‐strain‐1.10651096 [March 3, 2022]). According to statistics from Denmark as of February, Omicron BA.2 was responsible for 88% of all SARS‐CoV‐2 cases, and BA.2 may even evade BA.1‐induced natural immunity.^4^ Omicron BA.1‐BA.2 reinfections were found in 47 of the 187 (17%) reinfected samples. However, most of these cases were from individuals without a complete vaccination. BA.2 may trigger another wave of infection in areas that experienced BA.1, which was noteworthy.^5^ The Centers for Disease Control and Prevention (CDC) of America estimates that new cases of BA.2 leaped from one‐tenth of emerging COVID‐19 cases in the United States to nearly one‐quarter in 1 week (https://www.cdc.gov/coronavirus/2019‐ncov/index.html [July 15, 2022]). In addition, compared with BA.1, BA.2 could cause more severe diseases in hamsters.^6^ All the evidence suggests that the threat posed by BA.2 should not be underestimated.

The first diagnosis of Omicron was collected on November 9, 2021, and rapidly progressed from a 7‐day moving average of <10/100,000 to more than 25/100,000 in less than 10 days. In the early stages of the Omicron epidemic, Viana et al.^7^ estimated that it had a daily growth advantage of 0.24‐fold higher than Delta. South Africa suffered recurrent VOC assault and harbored around a quarter of vaccine‐elicited herd immunity. The frightening growth of Omicron in South Africa suggests a risk of breakthrough infection. According to a real‐world reinfection risk assessment in Qatar, previous infections conferred 90% protection against Alpha, Beta, and Delta but only 60% protection against Omicron.^8^ Ratio of the hazard for reinfections to the hazard for primary infections can indicate the risk of variant reinfection. The estimated relative hazard ratio of Omicron versus Alpha was 1.75, much higher than that of Delta (0.54) and Beta (0.71), which strongly hints the breakthrough infection ability of Omicron.^9^ Epidemic surveillance in several African countries has revealed that the Omicron possesses a faster and larger transmission rate than VOCs preceding it.^10^ Another study found that Omicron had a 36.5% higher transmissibility than Delta.^11^ Danish epidemic assessment revealed that the instantaneous reproduction number (R0) of Omicron is 3.19‐fold more elevated than that of Delta (R0 of Delta is 3.2–8, with an average of 5.0).^12^ Martin Hibberd, a professor of emerging infectious diseases at the London School of Hygiene and Tropical Medicine, estimates that the R0 of Omicron could be as high as 10.^13^ Two new subvariants BA.4 and BA.5 emerged in South Africa and were discovered in mid‐December 2021 and January 2022, respectively. The BA.4 and BA.5 Omicron subvariants also appeared in Europe in the following months.^14^ BA.4 and BA.5 Omicron subvariants have become new epidemic strains in many countries and spread more rapidly than other subtypes.^2^ Two reasons may account for their faster transmission. The first is that they have a stronger intrinsic transmission capacity versus the previous Omicron subvariants; the second is that BA.4 and BA.5 carry some key mutations that are more conducive to escaping the immune response.^2^ The key mutations F486V and L452R in the spike protein of BA.4/5 facilitate their immune escape at a faster rate and accelerate virus transmission.^15^, ^16^ BA.4/5 developed more significant escape responses to sera from those vaccinated with three doses COVID‐19 vaccine.^16^

### 2.2 The origin of Omicron

There are two main hypotheses regarding the source of Omicron; one is from the host with low immune function, and the other is from reverse zoonosis. Dr. Del Rio a distinguished professor of medicine in the Division of Infectious Diseases at Emory University School of Medicine believes that all these mutations occur in the same host and do not accumulate throughout the transmission.^17^ Molecular spectral analysis speculated that pre‐outbreak Omicron mutations are consistent with an evolutionary history in mice, not humans, suggesting the possibility of an early host jump in the origin of Omicron.^18^ Ins214EPE vanishing in previous mutations is presumably due to template switching caused by two or more coronaviruses coinfection in one host.^19^ Regardless of origin, adaptive mutations in the virus are evident. Thorne et al.^20^ hypothesized that mutations other than the spike protein might contribute to viral adaptation, which is supported by the significantly enhanced expression of natural immune antagonists (nucleocapsid protein (N), Orf9b, and Orf6) in Alpha. Simultaneously, comparable changes in the N and Orf9b regulatory regions from Omicron (28271A>T, 28881–28883GGG>AAC) suggested an adaptive viral transmission tendency and the importance of mutations outside the spike.^20^

### 2.3 The severity of the disease caused by Omicron infection

Preliminary data from South Africa^21^, ^22^ and England (https://spiral.imperial.ac.uk/handle/10044/1/93035 [December 22, 2021]) showed that people infected with Omicron are 40–45% less likely to require hospitalization than Delta. A survey by a hospital in South Africa showed that only one‐third of patients infected with Omicron developed pneumonia symptoms, 72% of pneumonia patients had mild to moderate disease, and 45% of patients needed oxygen supplementation. Clinical symptoms caused by Omicron appear to be diminishing compared with the Alpha.^23^ In Canada, Omicron has a 65% lower risk of hospitalization or death than Delta and an 83% lower risk of intensive care unit admission or death.^24^ Investigation in Norseland yielded similar results regarding the probability of hospitalization due to Omicron infection, which was decreased by 73%. It is worth noting that the reduction in risk of hospitalization for Omicron cases after two (Omicron: 66% vs. Delta: 93%) or three (Omicron: 86% vs. Delta: 88%) vaccinations was less than that of Delta.^22^, ^23^ These data suggest that Omicron was related to evading the vaccine protection and that the booster dose may be a stalling tactic during the pandemic. Compared with Delta, hospitalization duration for patients infected with Omicron was likewise declining.^25^ Studies find that people infected with Omicron tend to be younger.^23^, ^26^, ^27^ The Scottish report also mentioned that 48.9% of Omicron cases were between 20 and 39 years old (https://www.research.ed.ac.uk/en/publications/severity‐of‐omicron‐variant‐of‐concern‐and‐vaccine‐effectiveness [July 15, 2022]). In addition, children may be more susceptible to Omicron than variants preceding it. In the United States, children account for approximately 5% of all COVID‐19 hospitalization cases caused by Omicron, which is fourfold higher than the previous wave (Alpha, Beta, Delta),^28^ similar to South Africa.^29^ And this may be related to low vaccination rates among children. A study in England confirmed that vaccinated adults and children (12–17 years old) have significantly lower Omicron infection rates than unvaccinated children (5–11 years old).^30^ Fortunately, milder disease severity than predecessors were present in Omicron‐infected children, adults, and the elderly.^31^ An increase in asymptomatic rates of COVID‐19 may be caused by Omicron (https://www.who.int/publications/m/item/enhancing‐readiness‐for‐omicron‐(b.1.1.529)‐technical‐brief‐and‐priority‐actions‐for‐member‐states [January 21, 2022]). It is worth nothing that in areas ravaged by Omicron, most people have a history of SARS‐CoV‐2 infection and have received at least one dose of the COVID‐19 vaccine; therefore, milder disease severity does not indicate reduced virulence of Omicron, considering previous immunization could protect against serious diseases.

The clinical symptoms of Omicron vary owing to plentiful mutations. To study Omicron replication, Meng et al.^32^ revealed that although both Omicron and Delta could infect epithelial cells, Omicron viral replication was substantially lower in lower airway organs than Delta. Omicron presents an early infection advantage over Delta in human nasal epithelial cells.^33^ Compared with interferon‐active cells (caco‐2 and calu‐3), Omicron was more likely to infect the interferon‐deficient cells (Vero), implying a higher sensitivity of Omicron to interferon.^34^ In the hamster model, Omicron‐infected mice showed lower pulmonary infectivity and more serious pathogenicity than Delta and wild‐type (WT)‐infected mice,^35^ which was also confirmed in K18‐ACE2 mice.^36^ Preliminary research from the University of Hong Kong has shown that the infection and replication of the Omicron variant in the human bronchus are 70‐fold faster than that of its predecessor (https://www.the‐scientist.com/news‐opinion/omicron‐propagates‐70‐times‐faster‐than‐delta‐in‐bronchi‐study‐69540 [December 17, 2021]). Differences in the pattern of Omicron infection generated by abundant mutations were strongly associated with changes in the transmission rate and clinical severity. Omicron can enter cells effectively through the endosomal pathway independent of TMPRSS2 and is weak in inducing syncytial formation.^33^, ^35^, ^36^, ^37^, ^38^, ^39^ Variants preceding Omicron tended to adopt the TMPRSS2‐mediated membrane fusion route to infect host cells, but only a low proportion of upper respiratory tract cells expressed both ACE2 and TMPRSS2. This explains why Omicron infects the upper respiratory tract more frequently than its predecessors. It is worth mentioning that the change in the infective route broadens the range of cells that may be infected by Omicron and expands the damage caused by Omicron. In addition, a preference for the upper respiratory tract of Omicron makes it easier virus excretion through the nose and mouth, resulting in rapid transmission.

### 2.4 Mutations in spike protein of Omicron

**FIGURE 2:**
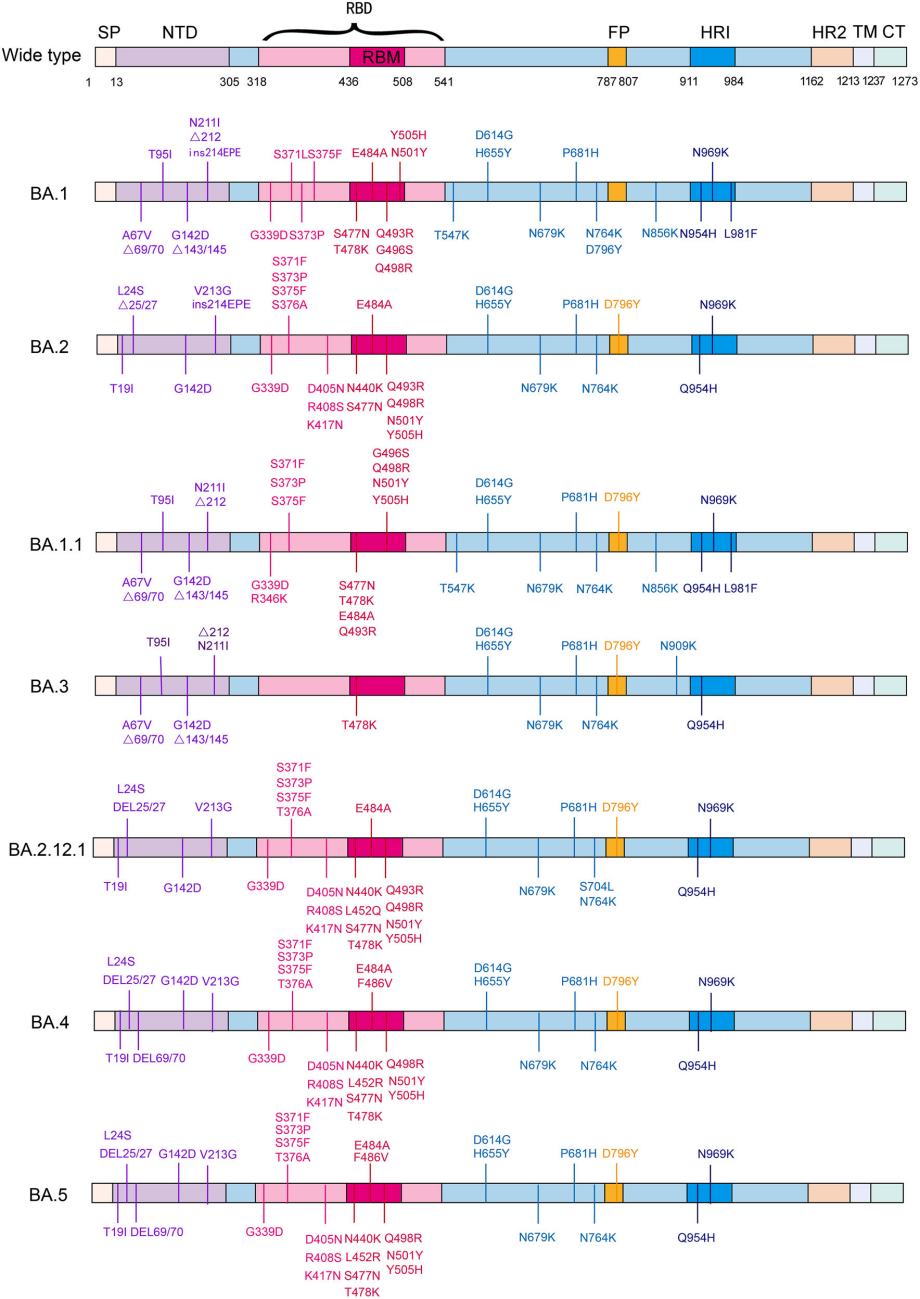
Mutations in Spike protein of seven Omicron subvariants. Schematic shows the locations of amino acid substitutions of seven Omicron subvariants (BA.1, BA.2, BA.1.1, BA.3, BA.2.12.1, BA.4, and BA.5) in spike protein. The RBD and RBM region is shown in shallow violet red and deep violet red respectively, and the N‐terminal domain (NTD) region is demonstrated in bluish violet. (The figure was drawn on “Adobe illustrator” tool)

Omicron carried a large number of mutations. The essential amino acid substitutions in spike protein are highlighted in red: A67V, del69‐70, T95I, del142‐144, Y145D, del211, L212I, ins214EPE, G339D, S371L, S373P, S375F, K417N, N440K, G446S, S477N, T478K, E484A, Q493R, G496S, Q498R, N501Y, Y505H, T547K, D614G, H655Y, N679K, P681H, N764K, D796Y, N856K, Q954H, N969K, and L981F. Fifteen of these were located in the receptor‐binding domain (RBD): G339D, S371L, S373P, S375F, K417N, N440K, G446S, S477N, T478K, E484A, Q493R, G496S, Q498R, N501Y, and Y505H (Figure 2).

The combination of Q498R and N501Y at the RBD/ACE2 interface (Q498R, N501Y, Q493R, Q498R, E484A, T478K, and S477N) has been shown to enhance binding affinity.^40^ Q498R is speculated to be responsible for the enhanced binding of Omicron RBD to mouse ACE2.^41^ This raises the alarm that Omicron can infect rodents and that the extension of the host range is harmful to human health in the long term. Deep mutation scan results of the remaining mutations in the RBD/ACE2 interface revealed no effect or negative consequence.^42^, ^43^, ^44^ The overall structure of Omicron spike protein may be more convincing for evaluating the binding relationship of the receptor. John et al.^41^ found that the binding affinity of Omicron RBD to human‐ACE2 was 2.4‐fold higher than that of WT by surface plasmon resonance. Cryo‐electron microscopic structural analysis of the Omicron variant spike protein complexed with hACE2 revealed that Q493R, G496S, and Q498R mutations could compensate for the weakened affinity caused by K417N.^45^ A peculiar RBD–RBD interaction induced by a ring consisting of S371L, S373P, G339D, and S375F mutations was identified in the Omicron S trimer structure but not in WT. It is expected to stabilize the upward conformation of RBD and facilitate its binding to ACE2.^46^ Han et al.^47^ analyzed the crystal structure of the RBD‐hACE2 complex and revealed that the affinity between the Omicron RBD and hACE2 was comparable to that of WT. Overall, the binding affinity between Omicron RBD and ACE2 was not superior to other VOCs (Delta, Beta). In addition to ACE2, the primary receptor, GRP78, a SARS‐CoV‐2 coreceptor, appears to have a stronger affinity for the Omicron spike protein than WT.^48^ Omicron pseudoviruses were found to infect HEK293T‐ACE2 cells easier than Beta, Delta, and D614G.^49^

**FIGURE 3:**
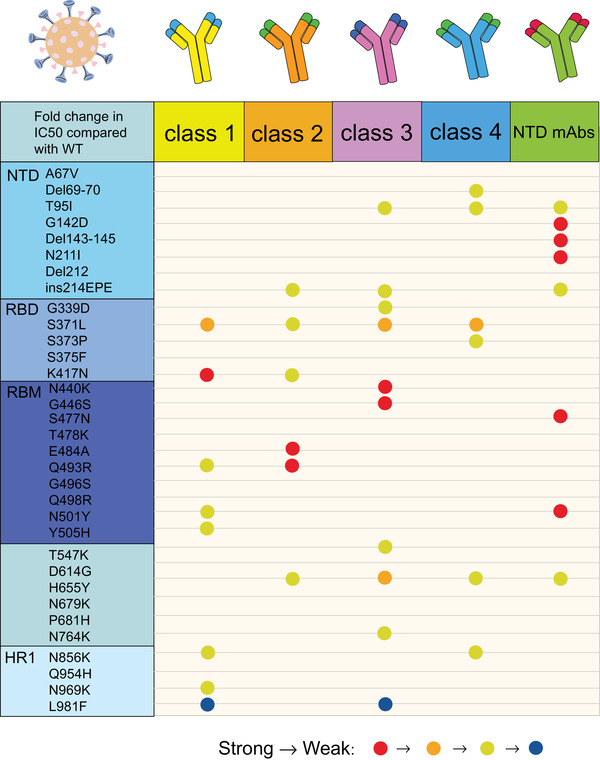
Resistance of individual mutations from Omicron spike protein to five types of antibodies (class 1, class 2, class 3, class 4, and NTD mAbs). The degree of resistance is represented by different colors. Resistance from strong to weak is indicated by red, orange, and yellow, respectively, while those favorable to antibody binding were blue. If the resistance strength is not marked, it indicates that there is little change in resistance to the antibody after the individual mutation. Mutations with strong resistance to NTD mAbs are G142D, Del143‐145, N211I, S477N, and N501Y; mutations with robust impedance to class 2 antibody are E484A and Q943R; L981F showed well binding ability to class 1 and class 3 antibodies. (The figure was drawn on “Adobe illustrator” tool)

Mutations in RBD are also responsible for viral evasion by clinical monoclonal antibodies. Of the five currently United States Food and Drug Administration (US FDA)‐approved emergency use authorization monoclonal antibody therapies (REGEN‐COV; a combination of Bamlanivimab and Etesevimab; Sotrovimab; Tocilizumab; Bebtelovimab), REGEN‐COV and the combination of Bamlanivimab and Etesevimab were eliminated as a treatment or postexposure prophylaxis for COVID‐19 due to the advent of Omicron (https://www.cms.gov/monoclonal [July 15, 2022]). Sotrovimab appears to be minimally affected by mutations in Omicron with a twofold to threefold neutralizing ability reduction, while the rest of the therapies are remarkably affected.^50^ Omicron generated tremendous leaps in resistance to all four classes of antibodies targeting the RBD (class 1, 2, 3, and 4) and antibodies targeting the N‐terminal domain (NTD) (Figure 3). E484A and Q493R influence the neutralizing activity of the class 2 antibodies, including LY‐CoV555 and 2–15 remarkably, leading to a loss of activity about 1000‐fold. The effect of N440K and G446S on the neutralizing activity of REGN10987 belonged to class 3; the newly emerged S371L in Omicron can even cause a decrease in the neutralizing activity of three classes of antibodies including class 1, class 3, and class 4.^50^ However, individual mutations do not always result in loss of binding or neutralization ability. Liu et al.^50^ used a pseudovirus neutralization model to assess the effect of five VOCs on antibodies. They confirmed that Omicron enables immune escape against more antibodies, developing resistance only against NTD antibodies and class 1 and 2, to almost complete resistance against class 1 and 2 RBD antibodies, and essential resistance against class 3 and 4 RBD antibodies.^50^

The proximity of H655Y, N679K, P681H, and D796Y to the furin cleavage site in the spike protein has been hypothesized to be associated with increased infectivity^51^; however, cellular‐level experiments have demonstrated these mutations may contribute to a weakening in facilitating S1/S2 cleavage.^33^, ^37^, ^38^, ^39^

The NTD contains four mutations, three deletions and one insertions: A67V, del69/70, T95I, G142D, del143/145, N211I, del212, and ins214EPE. Del69/70 also existed in Alpha and can be recognized by a useful detection method called s‐gene target failure (SGTF). BA.2 contains no 69/70 deletions and therefore cannot be detected by SGTF.

The numerous mutations resulting in altered local conformation, charge, and hydrophobic microenvironment of Omicron spike render them unrecognized by most NTD and RBD antibodies, leading to viral immune escape.^52^, ^53^ Interestingly, the accumulated mutations in Omicron included multiple essential amino acids (polar positively charged), increasing the total charge of the spike protein. This change may make spike protein more sensitive to low‐pH‐induced conformational changes.

## 3 DETECTION OF OMICRON VARIANT

Due to the large number of variants in Omicron, appropriate detection methods are urgently needed. The main detection methods for Omicron are standard polymerase chain reaction (PCR) assays, reverse transcription‐PCR (RT‐PCR) assays, multiplex qRT‐PCR assays,^54^ and viral genome sequencing.

Kozlov^55^ collected nasopharyngeal swabs from infected patients, and the viral load of different VOCs was measured by PCR technique. The results showed that patients infected with Delta had a higher peak viral load than those infected with Omicron.^55^ RT‐PCR is required for patients with positive samples of the Omicron variant. In addition, RT‐PCR testing is necessary for potentially infected individuals with Omicron, such as a history of recent travel to the epidemic area and close contact with confirmed patients.^56^, ^57^ For VOCs, including the Omicron variant, two‐tube multiplex qRT‐PCR can detect five copies of viral RNA.^58^ Next‐generation sequencing has become the gold standard for identifying Omicron. Scott et al.^59^ have shown that viral genome sequencing can be used for detecting apparent deletions of the S gene (S‐). Omicron mutation screening is another effective method for preventing the spread of the Omicron variant.^57^ Two single nucleotide polymorphism genotyping methods were used to screen the Omicron variant for the G339D or T547K mutation, which can also distinguish the Omicron variant from other VOCs.^60^ Furthermore, high‐resolution melting analysis methods can be used for screening Omicron and Delta variants.^61^ Besides, some antigen detection kits are helpful for rapid detection of Omicron in vitro.^62^ In addition, Omicron variants are also found in the wastewater of epidemic areas. An Omicron‐positive passenger was found on a flight from Johannesburg to Darwin, Australia, and the presence of Omicron variants in aircraft wastewater was confirmed, suggesting that aircraft wastewater may serve as an independent and invasive monitoring point.^63^ Coincidentally, the presence of Omicron variant was also found in outlet water at Frankfurt Airport in November 2021, even before the first clinical report of Omicron positivity in Germany on November 26, 2021.^64^ Similarly, Omicron was found in US community sewage samples from November to December 2021.^65^ Simultaneously, a novel SARS‐CoV‐2 lineage with multiple monoclonal resistances sharing many mutations with the Omicron variant was detected in sewage in New York City.^66^

## 4 OMICRON'S IMMUNE ESCAPE

Several studies have shown that the Omicron variant has significantly more immune evasion properties than its predecessor.^67^, ^68^ According to computational prediction, the Omicron variant can escape 85% of distinct epitopes from convalescent plasma and vaccine‐induced serum.^47^ Live virus neutralization experiments identified no neutralizing activity of Omicron in 16.5% of serum samples from recovering patients and vaccine recipients and a 14.5‐fold reduction in neutralizing titers compared with the WT in additional 85 serum samples.^69^ Omicron's immune evasion ability contributed to its rapid spread globally, and universal vaccine boosters can restore the protection to Omicron in a certain extent.^70^, ^71^ We systematically summarize the effects of Omicron on the immune responses induced by infection and vaccination (Table 1).

**TABLE 1:** Neutralization activity against Omicron of sera from different vaccinated individuals

Name	Reduced neutralization activity (compared with WT)	Positive proportion
BNT162b2 (two does)	5.9–122^50^, ^69^, ^72^, ^73^, ^74^, ^75^, ^76^, ^77^, ^78^, ^79^, ^80^, ^81^	12.8–45%^76^, ^77^, ^78^, ^81^, ^82^
mRNA1273 (two does)	4.8–43^50^, ^69^, ^79^, ^80^	not mentioned
ChAdOx1‐S (two does)	No neutralizing activity^50^, ^82^	4.5%^75^ (1/22) (28 days)
Ad26.COV2.S (two does)	No neutralizing activity^50^, ^83^	13.3%^83^ (2/15) (28 days)
CoronaVac (two does)	12.5^84^ or no neutralizing activity^77^, ^85^, ^86^	Not mentioned
BBIBP‐CorV (two does)	2.6–66.3^87^, ^88^, ^89^	2.7–25.68%^88^, ^89^, ^90^
ChAdOx‐1 S‐BNT162b2	No neutralizing activity^81^ or 14^91^	70%^91^
BNT162b2 (three does)	3.4–14.2^50^, ^69^, ^74^, ^80^, ^81^, ^91^	96.67–100%^78^, ^92^
mRNA‐1273 (three does)	6–16.7^69^, ^80^	Not mentioned
Inactivated vaccine (three does)	5.8–20.1^89^, ^90^, ^93^	78.08–95%^88^, ^93^
ADZ1222 (three does)	12.7^94^	Not mentioned
ZF2001 (three does)	9.4^95^	41.7%^95^

The table summarizes the proportion with detectable Omicron neutralization activity of serum from different vaccinated individuals and reduced neutralization activity against Omicron in serum samples from different vaccinated individuals compared with WT SARS‐CoV‐2.

### 4.1 Neutralization of Omicron in serum of convalescent patients

As expected, convalescent sera from COVID‐19 patients were resistant to various variants, with Omicron conferring the strongest immune escape.^96^ Analysis of the clinical data also revealed that the risk of reinfection with Omicron was higher than Delta in previous SARS‐CoV‐2‐infected patients.^97^ In vitro neutralization experiments found that patients infected with D614 virus had the most elevated antibody neutralization titers in serum approximately one month after infection. Then, the neutralization activity decreased over time.^98^, ^99^ Schmidt et al.^98^ found that the 50% neutralization titer (NT 50) value of Omicron was 58‐fold, 32‐fold, and 43‐fold lower than that of Wuhan‐hu‐1 after 1 month, 6 months, and 1 year of WT infection. In contrast, neutralizing activity against the Omicron variant was largely absent at day 300 or 12 months post‐WT infection.^78^, ^99^

Several independent teams have also successively demonstrated that only 0–47% of the convalescent serum samples from WT infected patients have neutralizing activity against Omicron.^50^, ^69^, ^76^, ^91^, ^95^, ^100^ Moreover, Liu et al.^101^ found that only serum from 22.2% (two out of nine) ICU and 11.1% (one out of nine) hospitalized patients have detectable Omicron neutralization antibody in D614G‐wave. At the same time, the antibody activity against Omicron showed a significant decrease compared with the WT, with 8.4–32 times^49^, ^50^, ^84^, ^95^, ^100^, ^102^ decrease in D614G convalescent serum and a 16.9‐fold reduction in the serum of patients infected with Victoria strain.^94^ Compared with the USA/WA1 strain, the Omicron‐neutralizing antibody titers were reduced by 15.8 and 4.4 folds in the serum collected from non‐Omicron‐infected patients after 1 and 6 months of WT infection, respectively.^103^ Similar results were obtained in enzyme‐linked immunosorbent assay (ELISA) experiments. The binding of Omicron spike protein to serum from WT infected patients was decreased by 4.4–8.3 times compared with that of WT spike protein, but this serum retained the binding to Omicron NTD, and the binding ability was reduced by 1.9 times compared with the WT.^69^

Whether SARS‐CoV‐2 variants can cause cross‐protection after infection is also an issue of widespread concern. Studies have found that the neutralizing activity of serum against Omicron is still limited after infection with most variants. The proportion of Omicron neutralizing activity from B.1.1.7 (Alpha) infected patients ranges from 0 to 40%,^76^, ^82^ and the neutralizing antibody titers against Omicron are reduced 18.4‐fold compared with that of Alpha variants (*n* = 18).^94^ Although Kimpel et al.^82^ found there was no neutralizing activity against Omicron in serum samples from patients with B.1.351 (Beta) infection, Weiss et al.^76^ found that two samples infected with Beta variants had neutralizing activity against Omicron above the threshold.

Sera from patients infected with the B.1.617.2 (Delta) variant appear to provide broader cross‐neutralization of Omicron; 0–85.7% of samples had detectable Omicron neutralization titers above the threshold.^76^, ^82^, ^90^, ^101^, ^104^ However, neutralization titers against the Omicron variants remained variably decreased in serum from Delta infected people, with a >26‐fold decrease compared with WT^90^ or 22.1–74.4‐fold lower than the Delta strain.^49^, ^76^ Even samples collected from people who recovered from 3 to 6 weeks after Delta infection only had good neutralization ability for USA/WA1 and Delta.^104^ However, some ICU patient samples collected during the Delta wave had neutralizing ability against Omicron comparable with those who received booster vaccines.^101^ A few studies have been conducted on individuals infected with the AY (AY.14/.25/.44/.47/.62/.74/.119) variant, and one report stated that 90% (nine out of 10) of the individuals infected with an AY variant had antibody titers above the threshold against Omicron.^76^

In addition, and somewhat unexpected, the cross‐neutralizing response induced by infection with the Omicron variants appeared to be limited. Ott et al.^104^ found that serum from mice infected with Omicron could only neutralize the Omicron variant itself, showing limited neutralization against the remaining variants, whereas sera from mice infected with the Delta showed good neutralizing activity against various variants, including Omicron (except the Beta variant).

### 4.2 Neutralization of Omicron by serum from two doses mRNA vaccine recipients

It is generally accepted that mRNA vaccination produces higher antibody‐neutralizing titers that peak around 28 days and decline significantly after 6 months, but also generate remarkably lower or even no neutralizing activity against Omicron.^105^ Vaccine efficacy against Omicron was 65.5% 2–4 weeks after the second dose of BNT162b2 and decreased to 15.4 and 8.8% after 15–19 and 25 weeks, respectively. The neutralizing activity against Omicron after two doses of mRNA‐1273 also reduced from 75.1% after 2 to 4 weeks to 14.9% after 25 or more weeks.^106^

Pfizer and BioNTech reported that the neutralization titer against the Omicron variant in the serum of individuals receiving two doses of their COVID‐19 vaccine decreased by more than 25 times.^94^ Taken together, the neutralizing antibody titers against Omicron in serum following two doses of mRNA vaccine (BNT162b2 or mRNA‐1273) were reduced about 21.3–30‐fold compared with the WT,^92^, ^98^, ^101^, ^107^ and 50–72.9% of these sera showed Omicron neutralization resistance.^92^, ^101^, ^107^ Some studies also showed that the neutralization activity from mRNA vaccinee's serum to Omicron decreased more in a short time after the second dose of mRNA vaccine, and the NT50 to Omicron of samples collected in 1.3 months after vaccination decreased 127 times than that of Wuhan‐hu‐1,^98^ while samples collected within 14–44 days after vaccination had little neutralization ability to Omicron variants.^108^

Studies have shown that the serum from mRNA‐1273 vaccine recipients appears to have slightly better neutralizing activity than that from Pfizer/BioNTech BNT162b2 vaccine recipients among healthcare workers (HCWs).^101^ Therefore, the effects of Omicron on mRNA‐1273 and BNT162b2 vaccines were compared and analyzed. Vaccinated versus unvaccinated participants were tested for neutralizing activity against BA.4 and BA.5 clinical isolates. In unvaccinated participants, FRNT50 against BA.4 and BA.5 decreased 7.6‐fold and 7.5‐fold compared with that of BA.1, respectively. In the vaccinated participants, FRNT50 against BA.4 and BA.5 decreased 3.2‐fold and 2.6‐fold compared with that of BA.1, respectively.^2^ The results indicated that the vaccinated people were more resistant to BA.4 and BA.5 than the unvaccinated people.^2^ Infection with BA.4 and BA.5 in vaccinated persons do not cause more severe clinical symptoms.^2^

The neutralization activity of serum to Omicron in patients vaccinated with two doses of BNT162b2 vaccine decreased about 5.9‐41.4 times compared with that of WT.^50^, ^69^, ^72^, ^73^, ^74^, ^75^, ^76^, ^77^, ^78^, ^79^ Surprisingly, Balazs et al.^80^ demonstrated that the neutralizing antibody activity against Omicron of the serum from BNT162b2 recipients had a 122‐fold decrease compared with WT after three months of vaccination by pseudovirus neutralization experiment. Meanwhile, only 12.8–45% of the samples vaccinated with two doses of BNT162b2 vaccine had detectable Omicron neutralizing activity,^76^, ^77^, ^78^, ^81^, ^82^ and the proportions of positive sera were only 20% and 24% against the HKU691 and HKU344‐R346K strains, respectively.^77^ Interestingly, the fold decrease in neutralizing activity of BNT162b2 vaccinees’ sera against Omicron over the WT appeared to diminish over time. Vaccine efficacy against Omicron decreased 37‐fold compared with the WT 0.5 weeks after the second dose of BNT162b2 and decreased 24.5‐ or 11.4‐fold after 3 or 6 months of the second dose of BNT162b2, respectively.^109^ A review of clinical data found that the severity of unvaccinated persons was five times when they were infected by Omicron than those who received two doses of Pfizer BNT162b2 mRNA vaccine.^110^ Comparable Omicron immune escape capacity was also observed in samples from recipients of two doses of mRNA‐1273 vaccine, with an average decrease in neutralization titers of approximately 4.8–43‐fold.^50^, ^69^, ^79^, ^80^

In conclusion, there seems to be no significant difference in the neutralization effect against Omicron between the serum from people who received two doses of mRNA‐1273 and BNT162b2 vaccinees. In addition, WT and Omicron variant RBD binding did not differ significantly between the two doses of mRNA‐1273 or BNT162b2 vaccinated sera in the ELISA experimental group. The binding ability between Omicron RBD and the sera vaccinated with two doses of mRNA‐1273 or BNT162b2 vaccine was 2.9 times and 1.5 times lower than that of WT RBD and these sera, respectively.^69^

### 4.3 Neutralization of Omicron by two doses adenovirus vaccine recipients

Relevant studies have shown that the adenovirus vector vaccine ChAdOx1‐S/nCoV‐19 and Ad26.COV2.S had significantly less neutralizing activity than the mRNA vaccine at 28 and 56 days postvaccination.^105^ However, the neutralizing antibody in serum from people vaccinated with a two doses adenovirus vaccine seems to be more stable and not easily decrease over time.^105^ Meanwhile, the protective effect against Omicron in the serum of adenovirus vector vaccine recipients is minimal or even no neutralization effect has been found in most studies. No neutralizing activity against Omicron was found in serum 1 month or 91–159 days after receiving two doses of the ChAdOx1‐S/nCoV‐19 vaccine,^50^, ^82^, ^106^ or only one of 22 samples (28 days after vaccination) had neutralization activity against Omicron.^75^ A similar situation occurred with two doses of the Ad26.COV2.S vaccine, with no neutralizing activity against Omicron in samples 50–186 days after vaccination,^50^, ^105^ or only 13.3% of samples had Omicron variant neutralization activity 28 days after vaccination.^105^

### 4.4 Neutralization of Omicron by serum from recipients receiving two doses of inactivated virus vaccine

Inactivated vaccines are currently the most widely used COVID‐19 vaccines, but they may have minor protection against Omicron infection.^111^ For the CoronaVac vaccine, almost no detectable neutralizing antibody titers against Omicron in recipient sera or very low antibody titers can be detected.^77^, ^85^, ^86^ However, Wang et al.^84^ found detectable neutralizing activity against Omicron in samples collected 14 days after two doses of Corona Vac vaccine, with a 12.5‐fold decrease in neutralization activity compared with the WT. The proportion of Omicron positive serum in recipients of the BBIBP‐CorV vaccine from Sinopharm is only 2.7–25.68%.^88^, ^89^, ^90^ And the neutralization titer against Omicron of serum vaccinated two doses of BBIBP‐CorV vaccine decreased by approximately 2.6–66.3 times compared with the WT.^87^, ^88^, ^89^ Neutralizing activity against Omicron decreased by only 2.6‐fold at 4–8 months after two doses of inactivated vaccine, but this appeared to correlate with a large decrease in neutralizing titers of WT. On the 14th day after two doses of inactivated vaccination, the neutralizing activity decreased by approximately 11.16–20 times.^87^, ^89^ On the 28th day after vaccination, it fell by 66.3 times compared with the WT.^88^

### 4.5 Heterologous inoculation

Heterologous vaccination is uncommon in two doses vaccinated populations, and some studies showed that heterologous vaccination seem to enhance neutralizing activity against Omicron. Sera collected within one month after vaccination with heterologous ChAdOx1‐S/BNT162b2 showed higher neutralizing activity than sera collected within three months after vaccination with BNT162b2/BNT162b2.^91^ The inhibition efficiency against Omicron of the sera from ChAdOx1‐S/BNT162b2‐vaccinated individuals was 14 times lower than that of D614G,^91^ and the positive rate against Omicron in these serum samples was 14 out of 20.^82^ However, it was also reported that the serum from heterologous ChAdOx1‐S/BNT162b2 vaccine recipients displayed no neutralizing response to Omicron.^109^

### 4.6 Neutralization of Omicron after vaccination in convalescent patients

Studies have shown that the revaccination of patients infected with SARS‐CoV‐2 can significantly increase the neutralizing activity against Omicron. Nine out of ten samples that received a single dose of vaccine following infection had Omicron neutralizing activity.^82^ Meanwhile, the neutralization capacity against the Omicron variant of individuals who received two doses of BNT162b2 vaccine after SARS‐CoV‐2 infection is comparable to that against the D614G.^112^ Although the sera of postinfection and completion of three doses of mRNA vaccination populations had considerable neutralization activity against Omicron, it is undeniable that its neutralizing activity against Omicron had decreased to varying degrees compared with the WT.^113^ A single dose of BNT162b2 induced a substantial increase in neutralizing activity against Omicron of convalescent individuals’ serum, with a GeoMean ID 50 was 1549, and 5.16 times lower than that of WT.^78^ Two additional doses of BNT162b2 or mRNA‐1272 increased the neutralizing activity against Omicron of convalescent individuals’ serum, by 12–32.8 times.^69^, ^80^, ^109^ The neutralizing activity against Omicron of serum from convalescent individuals vaccinated with Ad26.COV2.S was 17‐fold lower than the WT.^80^ The three doses vaccination further improved the neutralizing activity against Omicron, with an average increase of two to three times the neutralizing titer,^100^ which was only 1.54 times lower than the WT.^98^

### 4.7 The neutralizing effect against Omicron after the booster injection

Based on current evidence, it is widely believed that a booster dose against COVID‐19 provides further protection to vaccinated individuals and that booster doses are critical regardless of the circulating variant.^57^ Arashiro et al.^114^, ^115^ believe that strengthening vaccination is still the best option against Omicron in Japan. Moreover, additional doses of the vaccine significantly increased the number of neutralizing antibodies in the serum from the vaccinated population,^116^ but further clinical data are still needed to determine the efficacy of boosters.^117^ Booster doses of both mRNA‐1273 and CoronaVac/PiCoVacc, and other mRNA‐based vaccines, were potent and effective in causing sustained enhancement of Omicron neutralization 6 months after the second vaccination,^113^ which increased the neutralization ability approximately between threefold and 133‐fold.^87^ Although Omicron has a significant immune evasion ability in almost all sera from recovered patients or two doses COVID‐19 vaccinees, the reduction in neutralization ability under the effect of booster is only four to eight times.^118^

Clinical data have also shown that the booster dose reduces the infection risk and morbidity ratio of Omicron to a certain extent. The CDC stated that on January 21, 2022, the third dose of the mRNA vaccine prevented 82% of patients infected with Omicron from entering the emergency room or urgent care and 90% from being hospitalized.^119^ Without a booster shot, unvaccinated cases were 41% more likely to infect other family members than fully vaccinated cases.^120^ In October–November 2021 period, the incidence rate ratio (IRR) of the booster vaccinated group was 4.9, and the IRR of group without a booster was 2.5 in the United States, and the booster significantly affected Omicron infection and death caused by SARS‐CoV‐2 in people aged 50–64 years.^121^ However, data from the UK showed that 10 weeks after the third injection, the effectiveness of the booster against hospitalization dropped from 92 to 83%, and the protection provided by the booster appeared to be waning.^119^ Thus, an ongoing comprehensive assessment of the effects of boosters is needed.

#### 4.7.1 Effects of homologous reinforcement

The neutralizing antibodies against Omicron had a significant (20–30‐fold) increase a few months later with a third dose of the same vaccine boost, but only a modest (onefold to fourfold) increase against the WT.^119^

##### mRNA vaccine homologous boost

Overall, the mRNA vaccine booster substantially boosted the neutralizing activity of the recipients’ sera, and the neutralizing activity was still largely retained over time. However, the neutralizing antibody titers against Omicron were still lower than the WT. Vaccine effectiveness increased to 67.2% 2–4 weeks after the third BNT162b2 booster dose, with 45.7% remaining after 10 weeks or longer. And after the mRNA‐1273 homologous boost for 2–4 weeks, vaccine effectiveness reached 66.3%.^106^ The neutralizing antibody titers of serum from mRNA homologous booster‐vaccinated individuals against Omicron pseudovirus or live virus improved approximately 4.1‐ to more than 100 fold.^50^, ^74^, ^78^, ^81^, ^91^, ^94^, ^108^, ^122^ Three doses of BNT162b2 increased infection protection by 10‐fold,^91^ with an average of >4.1‐fold increase in neutralizing activity against Omicron 14–90 days after booster vaccination.^50^

Approximately 1 month after mRNA booster vaccination, the neutralizing activity against the Omicron live virus of serum was 17.6‐fold higher than that of the two doses of mRNA vaccinees’ serum. And the neutralizing activity of serum from mRNA booster vaccinees against the Omicron pseudovirus increased by 23.4‐fold compared with that against the WT from the second dose of mRNA vaccinees.^81^ Similarly, the mRNA homologous booster improved the neutralizing activity against Omicron about 15–100 times over 14–44 days after the boosting.^94^, ^98^, ^108^ In addition, the breadth of protection induced after the booster was also greatly increased, essentially with the proportion against Omicron positive serum samples above 90% and even 100%.^50^, ^78^, ^81^, ^107^ However, at the same time, compared with the WT, the neutralization activity against Omicron still had a slight decrease, with an average reduction of about 3.3–14 times.^50^, ^81^, ^101^, ^107^ Three doses of BNT162b2 resulted in the neutralization activity of the serum against Omicron was 14.2‐fold lower than that against Victoria.^94^ Some even reported that serum neutralizing activity against the Omicron variant after the BNT162b2 vaccine booster is greater than that against Wu‐01.^78^ The neutralizing activity against Omicron was slightly different in the serum from BNT162b2 or mRNA‐1273 homologous booster vaccinated people. The neutralizing activity against Omicron was 7.5 or 16.7 times and four or six times lower than that against the WT in the BNT162b2 booster or mRNA‐1273 booster, respectively.^69^, ^80^ All together, BNT162b2 booster may show slightly higher neutralizing antibody titers against Omicron than the mRNA‐1273 vaccine booster, and the same finding was also found in HCWs.^101^ Statistical analysis yielded similar results, the adjusted odds ratio (ORs) for Omicron were 0.35 for three doses of BNT162b2 versus unvaccinated and 0.28 for three doses of mRNA‐1273 versus unvaccinated. And the adjusted ORs for Omicron were 0.35 for three doses of BNT162b2 versus two doses and 0.31 for three doses of mRNA‐1273 versus two doses.^117^

##### Adenovirus vaccine homologous boost

The three doses of ADZ1222 vaccine increased 2.7‐fold than the second dose in the serum neutralization activity against Omicron 28 days after vaccination, although the neutralization activity against Omicron was 12.7 times lower than the Victoria.^94^ While the Ad26COV2.S booster resulted in the neutralizing activity of the serum against Omicron was approximately 13 times lower than that against WT.^80^

##### Inactivated vaccine homologous boost

Similarly, the inactivated vaccine homologous booster provided more protection to Omicron to a certain extent, and three doses of inactivated vaccine significantly improved the neutralization effect against Omicron, with 46.7% effectiveness against Omicron at 5–9 weeks after inactivated booster vaccination^106^ and an average seroconversion rate of 78.08–100%.^76^, ^80^, ^93^, ^99^ In a study with a seroconversion rate of 95% (57 out of 60), the seroconversion rate of inactivated homologous booster vaccination was 91.7% higher than that of two doses vaccination.^93^ However, only 35% of patients were seropositive for Omicron 28 days after the CoronaVac booster.^85^ At the same time, three doses of inactivated vaccine will increase the neutralization titer against Omicron about 3.3–31.8 times than that of two doses of inactivated vaccine,^73^, ^76^, ^88^, ^89^, ^90^ and the titer was almost equal to or even higher than that observed 7 days after the second dose.^99^ The neutralization antibody titers against Omicron of serum from inactivated boosters were still lower than against other variants^99^ and 5.8–20.1 times lower than against the WT.^88^, ^89^, ^93^

##### Subunit virus vaccine boost

In individuals vaccinated with the RBD‐based protein subunit vaccine ZF2001, 58.3% (seven out of 12) of sera sampled 15–60 days after the third dose could not neutralize Omicron. And the geometric mean titers (GMT, 50% inhibitory dose [ID 50]) of these sera against Omicron were 9.4‐fold lower than those against D614G.^95^

#### 4.7.2 Effects of heterologous boosting

Previous studies have found that heterologous booster seem to induce a stronger, longer‐lasting immune response.^123^ However, for the Omicron variant, heterologous boosting did not appear to have a significantly enhanced effect over homologous boosting. Two doses of BBIBP‐CorV vaccination followed by subunit vaccine ZF2001 boosters increased neutralizing titers by more than 45‐fold for WT and approximately fourfold for Omicron.^90^ The neutralization activity against Omicron increased 1.4‐fold in the serum vaccinated with the BNT162b2 vaccine as a booster following two doses of CoronaVac vaccine, although it was still reduced 7.1‐fold compared with the WT^86^; the BBIBP‐CorV/ZF2001 heterologous booster increased the neutralization activity by 5.16 times of the serum collected 14 days after vaccination and 5.85 times 28 days after vaccination compared with that before the booster.^89^ Similar to the homologous boost, the neutralizing activity against Omicron after the heterologous boost was still lower than that of the WT. The neutralization titer of the serum vaccinated with BBIBP‐CorV/ZF2001 was 14.9 times lower than that of the WT 14 days after the heterologous boost, and 7.3 times lower than that of the WT 28 days after the heterologous boost^89^; BNT162b2 booster vaccine partially restored neutralization against the Omicron variant after two doses of mRNA‐1273 or Ad26.COV.2, and the neutralization activity against Omicron of serum from individuals vaccinated with BNT162b2 booster had a 17‐fold reduction compared with WT^105^; serum neutralization capacity against Omicron from individuals vaccinated with BNT162b2 as a booster after two doses of mRNA‐1273 vaccine increased to 64.9%, but also 20 and 22.7 times lower than the WT at 6 months or 0.5 months after vaccination; whereas mRNA‐1273 booster vaccination increased vaccine efficacy to 73.9% after a two doses BNT162b2, then decreased to 64.4% after 5–9 weeks; ChAdOx1‐S/nCoV‐19 inoculated as the first two doses, BNT162b2 and mRNA‐1273 booster doses increased their neutralizing activity against Omicron to 62.4 and 70.1% after 2–4 weeks, respectively, and then dropped to 39.6 and 60.9%.^106^, ^109^ Serum neutralization capacity against Omicron in BNT162b2‐enhanced mice after two doses of mRNA‐1273 vaccination has also increased and was 22.7‐fold lower than that against the WT.^79^ IgG antibody concentrations increased significantly, and 76% of samples were sensitive to Omicron in all groups 28 days after the heterologous booster dose following two previous doses of the CoronaVac inactivated virus vaccine. And the seropositivity ratios of Ad26.CoV2.S, BNT162b2, and ChAdOx1‐S/nCoV‐19 as heterologous booster vaccination reached 90%.^85^

### 4.8 Effect of breakthrough infection on Omicron neutralization after vaccination

Omicron breakthrough infection after vaccination also further increased the level of neutralizing antibodies in serum, with binding antibody titers of samples from Omicron breakthrough individuals (three doses of BNT162b2 vaccine (*n* = 6), two doses of BNT162b2 vaccine followed by a booster shot of CX‐024414 or ChAdOx1‐S‐BNT162B2 vaccine) were comparable to that four weeks after the second dose of vaccine.^124^ The Omicron breakthrough infection (OP1) occurred 178 days after the second dose of BNT162b2 and induced the neutralizing activity of serum from <1:20 to an average IC 50 value of 1:2929, with 74.89‐fold higher than in those who received only two doses of BNT162b2; While the mean IC50 value against Omicron in the serum of patients with confirmed Omicron infection (OP2) 53 days after vaccination with mRNA‐1273 was 1:854.5.^72^ And sera from vaccinated individuals with confirmed Omicron breakthrough infection showed the highest level of protection (>80%) against most variants, including WA1, Delta, Alpha, Beta, and Omicron.^104^ Breakthrough infection with other variants can also significantly increase the protective activity of Omicron. The serum from individuals with breakthrough infection of the Delta variant has a neutralizing effect against many variants, including Omicron, but the NT50 of Omicron is relatively low.^104^ The GMT against the Omicron variant and WT in serum from patients with Delta breakthrough infection after two doses of inactivated vaccine (CoronaVac) were 154.00 and 1794.40, and the neutralizing activity against Omicron was an 11.65‐fold reduction compared with the WT.^89^ Neutralizing antibody activity against Omicron could be detected in nine of ten serum samples from the individuals with two doses vaccination followed by breakthrough infection or convalescent participants followed by two doses vaccination, although the neutralizing activity was lower than that of the Delta variant.^82^

### 4.9 The effect of Omicron infection on cellular immunity

T‐cell immune responses elicited by infection with SARS‐CoV‐2 or vaccines are still effective against Omicron, and SARS‐CoV‐2 has not yet evolved extensive T‐cell escape mutations.^125^ Eight months after two doses of Ad26.COV2.S vaccination, the spike protein‐specific CD8^+^ T cell responses in serum against WA1, Delta, and Omicron strains were 0.061, 0.062, and 0.051%, respectively, and the specific CD4^+^ T cell responses were 0.026, 0.030, and 0.029%, respectively. And the serum from individuals vaccinated with two doses of BNT162b2 had 0.028 and 0.023% CD8^+^ T cell responses against WA1 and Omicron, and 0.033 and 0.027% CD4^+^ T cell responses, respectively. Cellular immunity induced after vaccination remained highly cross‐reactive to Omicron variants.^126^ The capacity of T cells to respond to Omicron spike protein was maintained at 70–80% in participants vaccinated with Ad26.CoV2.S, BNT162b2, or unvaccinated convalescent COVID‐19 patients (*n* = 70), and T cell responses induced by vaccination or infection could still recognize the Omicron variant.^127^, ^128^

### 4.10 Antibody activity against Omicron in special populations

The neutralization activity of serum from lung cancer patients vaccinated with mRNA vaccine showed lower than that from healthy adults vaccinated with the same type of vaccine according to live virus neutralization experiments. At the same time, there was no significant difference in the antibody response to SARS‐CoV‐2 vaccination in patients receiving either PD‐1 monotherapy or a combination of chemotherapy and PD‐1 targeted therapy compared with patients receiving no cancer therapy at the time of vaccination. Similarly, the neutralizing activity against Omicron in the serum of lung cancer patients after receiving the mRNA vaccine was 79‐fold lower than that of the WT.^129^ Pseudovirus neutralization experiments found that 4 weeks after the second dose of mRNA‐1273 (100 μg), the GMT against Omicron of serum was 28.8‐fold lower than that against D614G in adults over 18 years; 11.8‐fold lower than that against D614G in adolescents (12–17 years), and the GMTs against D614G and Omicron variants in adolescents were 1.5‐fold and 3.8‐fold higher than that in adults, respectively. Similarly, the GMTs against Omicron were 22.1‐fold lower than those against D614G in children (6–12 years old), and the GMTs against D614G and Omicron in children were 2.0 and 2.5 times higher than those that in adults, respectively.^130^ Among those who received two doses of the BNT162b2 vaccine with a mean age of 15 years, only 38.2% (13 out of 34) had detectable Omicron neutralizing titers, and their GMT was 7.5, which was 20‐fold lower than that of the WT (GMT = 150.5). In a population of COVID‐19 recovered patients with an average age of 9.6 years, 26.7% (four out of 15) of the serum samples were able to neutralize the Omicron variant, and its GMT value was only 6.3, which was 20 times lower than that of the WT (GMT = 127).^131^ Compared with D614G, the neutralization titer against the Omicron variant was only 5.1‐fold lower in the sera of cancer patients following the mRNA vaccine booster, and 88.9% (24 out of 27) of the patients had Omicron neutralization activity.^92^

Vaccination remains significantly associated with a substantial reduction in the risk of symptomatic COVID‐19, both during and before the Omicron wave.^132^ Moderna, Pfizer, and their partner BioNTech announced that boosters tailored for Omicron may offer better protection and they could supply Omicron‐tailored vaccines as early as March 2022, although moving to a new vaccine would reduce current vaccine production. John Moore, an immunologist at Weill Cornell Medical College, said that the Omicron wave might be over when these vaccines are scaled up. The development of a universal vaccine effective for all SARS‐CoV‐2 variants instead of frequently updating the vaccines for the emerging variant is promising and expected.^70^, ^133^ Humans repeatedly exposed to the SARS‐CoV‐2 spike protein through infection or booster doses are likely to have neutralizing antibody activity against Omicron. Moreover, studies have also shown that even booster doses are insufficient to prevent symptomatic disease and emphasize the need to maintain additional non‐pharmacological interventions.^117^

## 5 PROPHYLAXIS AND TREATMENT OF OMICRON VARIANT

### 5.1 Drug treatment of Omicron variants

The emergence of various variants during the COVID‐19 pandemic has created a great challenge for the development of vaccines. Antiviral drugs have more stable chemical structures compared with vaccines. The sensitivities of the Omicron to antiviral drugs are summarized in Table 2. Effective drugs are always important for treating COVID‐19 and helping to end global epidemics.^134^

**TABLE 2:** Sensitivities of the Omicron (B.1.1.529) to different antiviral drugs

Antiviral	Viral lineage	Viral type	Full/partial variant	Fold change	Reference strain
Camostat	B.1.1.529^135^	Live virus	Full variant	1.54	B.1.617.2
Molnupiravir	B.1.1.529^136^	Live virus	Full variant	0.5	Wuhan‐Hu‐1
Remdesivir	B.1.1.529^137^	Live virus	Full variant	0.58	A
Ensovibep	B.1.1.529(BA.1)^138^	Pseudovirus	Full variant	3.27	Wuhan‐Hu‐1
GS‐441524	B.1.1.529^139^	Live virus	Full variant	0.77	USA‐WA1/2020
Nirmatrelvir	B.1.1.529^135^	Live virus	Full variant	2.32	B.1.617.2
S‐217622	B.1.1.529^137^	Live virus	Full variant	0.78	A

“Fold change” is an indicator of susceptivity of Omicron to potential antiviral drugs.

Remdesivir, a viral RNA‐dependent RNA polymerase (RdRp) inhibitor, has been approved by the US FDA for the emergency treatment of COVID‐19, which inhibits the replication of several RNA viruses, including coronaviruses.^140^ Molnupiravir, a derivative of the antiviral drug ribavirin, the active ingredient of which is EIDD‐1931, was first approved in the United Kingdom for the treatment of SARS‐CoV‐2‐infected patients.^34^, ^141^ Several studies have confirmed the effectiveness of remdesivir and molnupiravir against Omicron BA.1.^32^, ^37^ Remdesivir, molnupiravir, and the viral protease inhibitor nirmatrelvir are highly conserved in their target proteins, which have the same antiviral activity against the original strains and VOCs (Alpha, Beta, Gamma, Delta, and Omicron).^136^ Li et al.^142^ used clinical isolates cultured from infected patients to assess whether molnupiravir had inhibitory effects on Omicron variants. Viral replication was significantly inhibited by molnupiravir in SARS‐CoV‐2‐infected cells. Omicron replication could also be inhibited by nirmatrelvir in Calu‐3 cells,^142^ and combined treatment with molnupiravir and nirmatrelvir showed synergistic antiviral activity against both WT and Omicron.^142^ The oral novel COVID‐19 therapeutic drug PF‐07321332 (Pfizer) is a ritonavir‐boosted protease inhibitor. Drożdżal et al.^143^ studied COVID‐19 patients (non‐hospitalized adults) in a randomized double‐blind controlled study, patients received PF‐07321332 treatment within 3 days of symptom onset, and the results showed that the risk of hospitalization and death associated with SARS‐CoV‐2 was reduced by 89%. PF‐07321332, ritonavir, and paxlovid effectively reduced the hospitalization duration of COVID‐19 infected patients in clinical trials.^140^ Potent oral antiviral drugs, such as molnupiravir and PF07321332, could avoid side effects such as phlebitis caused by intravenous medications and have shown better clinical outcomes.^113^, ^140^

Corticosteroids, such as dexamethasone, were used in critically ill patients infected with the Omicron variant, especially in patients on high‐flow oxygen therapy, noninvasive ventilation, and mechanical ventilation. However, dexamethasone should not be recommended for mild to moderate COVID‐19 patients (in patients not receiving oxygen therapy).^140^ In addition, hydrocortisone, methylprednisolone, and prednisone were equally effective in patients infected with Omicron variants. Corticosteroids have been shown to have satisfactory anti‐inflammatory and immunomodulatory effects on Omicron infection.^140^

Targeted anti‐inflammatory drugs, such as IL‐6 and JAK inhibitors, can inhibit the Omicron variants. Tocilizumab effectively reduces inflammation and mortality in patients.^144^ Whether sarilumab is effective against Omicron variants still needs further studies in the future. Tocilizumab and baricitinib were probably effective in severe cases of Omicron infection.^57^

Both WHO and related studies have indicated that JAK and IL‐6 inhibitors in combination with dexamethasone contribute to remission in patients with Omicron infection.^34^, ^115^ Bafilomycin A1 and chloroquine effectively suppressed Omicron variants.^145^ Eight drugs (PF‐07321332, EIDD‐1931, ribavirin, nafamostat, favipiravir, remdesivir, aprotinin, and camostat) from the antiviral assay were used on the Omicron and Delta isolates, which were found to be similarly susceptible to both variants, with the Omicron remaining sensitive to broad‐spectrum anti‐SARS‐CoV‐2 drugs.^34^ Adequate protection of the respiratory tract from Omicron infection (B.1.351 and B.1.1.529) was achieved by nasal administration of interferon‐λ in mice.^146^ Some studies have demonstrated that hydroxychloroquine, convalescent plasma, and camostat were ineffective in treating patients infected with SARS‐CoV‐2 Omicron variants. However, amantadine, the effects of ivermectin and clonidine on Omicron infection were uncertain, and further studies are needed in the future.^143^, ^145^

### 5.2 Neutralizing antibody

#### 5.2.1 Protective effects of monoclonal antibodies against Omicron variant

The Omicron variant contains a wide range of mutations that can escape neutralizing antibodies produced from vaccination. Nevertheless, monoclonal antibodies have the merits of high biotransformation efficiency, high specificity, high immunogenicity, and transparent mechanism of action.

Compared with previous seasonal coronaviruses and influenza A, SARS‐CoV‐2 has faster evolutionary and propagation rates.^147^ Most monoclonal antibodies induced by the receptor‐binding domain (RBM) have lost neutralizing antibody activity against Omicron, whereas some monoclonal antibodies recognize Omicron by antigenic binding sites outside the RBM. The neutralizing antibody recognizes the conserved RBD epitope of VOCs, which is beneficial for constraining VOCs infection.^41^ Hence, the sensitivities of the Omicron to different neutralizing antibodies are indicated by “fold change” in Table 3.

**TABLE 3:** Sensibilities of the Omicron (B.1.1.529) to diverse neutralizing antibodies

neutralizing antibody (NAB)	Viral lineage	Viral type	Full/partial variant	Fold change	Reference strain
Amubarvimab	B.1.1.529^148^	Pseudovirus	Partial variant	14.4	Wuhan‐Hu‐1
	B.1.1.529^148^	Pseudovirus	Partial variant	42.1	Wuhan‐Hu‐1
	B.1.1.529(BA.4/5)^149^	Pseudovirus	Full variant	134.42	Wuhan‐Hu‐1 D614G
Bamlanivimab	B.1.1.529^148^	Pseudovirus	Partial variant	291	Wuhan‐Hu‐1 D614G
Bebtelovimab	B.1.1.529^148^	Pseudovirus	Partial variant	3.5	Wuhan‐Hu‐1
	B.1.1.529(BA.4/5)^149^	Pseudovirus	Full variant	1.29	Wuhan‐Hu‐1 D614G
Casirivimab	B.1.1.529^150^	Pseudovirus	Partial variant	27.78	B.1
	B.1.1.529^151^	Pseudovirus	Partial variant	833.33	Wuhan‐Hu‐1
Cilgavimab	B.1.1.529^50^	Pseudovirus	Full variant	390	USA‐WA1/2020 D614G
	B.1.1.529^152^	Live virus	Full variant	268.31	VIC
	B.1.1.529(BA.4/5)^153^	Pseudovirus	Full variant	25.35	B.1.1
Etesevimab	B.1.1.529^148^	Pseudovirus	Partial variant	3	Wuhan‐Hu‐1
	B.1.1.529^154^	Live virus	Full variant	138.89	USA‐WA1/2020
	B.1.1.529^148^	Pseudovirus	Partial variant	630	Wuhan‐Hu‐1
Evusheld	B.1.1.529^151^	Pseudovirus	Partial variant	262.2	Wuhan‐Hu‐1
	B.1.1.529(BA.4/5)^149^	Pseudovirus	Pseudovirus	19.05	Wuhan‐Hu‐1 D614G
Imdevimab	B.1.1.529^148^	Pseudovirus	Partial variant	103	Wuhan‐Hu‐1
	B.1.1.529(BA.4/5)^149^	Pseudovirus	Full variant	91.23	Wuhan‐Hu‐1 D614G
Regdanvimab	B.1.1.529^155^	Pseudovirus	Full variant	>=1000	Wuhan‐Hu‐1
Romlusevimab	B.1.1.529^50^	Pseudovirus	Full variant	0.45	USA‐WA1/2020 D614G
	B.1.1.529(BA.4/5)^149^	Pseudovirus	Full variant	8.07	Wuhan‐Hu‐1 D614G
Ronapreve	B.1.1.529^156^	Pseudovirus	Full variant	>=1000	Wuhan‐Hu‐1
	B.1.1.529(BA.4/5)^149^	Pseudovirus	Full variant	141.8	Wuhan‐Hu‐1 D614G
Sotrovimab	B.1.1.529^157^	Live virus	Full variant	15.56	USA‐WA1/2020
	B.1.1.529^41^	Pseudovirus	Full variant	3	Wuhan‐Hu‐1
	B.1.1.529(BA.4/5)^153^	Pseudovirus	Full variant	13.41	B.1.1
Tixagevimab	B.1.1.529^158^	Pseudovirus	Full variant	73.8	A.2.2
	B.1.1.529^151^	Pseudovirus	Partial variant	>=1000	Wuhan‐Hu‐1
VIR‐7832	B.1.1.529^41^	Pseudovirus	Full variant	3	Wuhan‐Hu‐1

“Fold change” is an indicator of sensibility of Omicron to dissimilar neutralizing antibodies.

Kovacech et al.^159^ showed that AX290 and AX677 have a nanoscale affinity to the RBD of the viral spike protein. Sotrovimab binds conserved epitopes of the RBD, which were used as a cocktail therapy for Omicron infection.^160^ GlaxoSmithKline's VIR‐7831 (sotrovimab) and Regeneron monoclonal antibody cocktail have shown better efficacy.^161^, ^162^ VIR‐7831 (sotrovimab) and VIR‐7832 are dual‐acting monoclonal antibodies against spike‐in glycoproteins with neutralizing activity against Omicron variants.^57^ Moreover, sotrovimab is more neutralizing against Omicron variants.^91^ It has been shown that Hu33 has a higher neutralizing effect against Omicron pseudovirus than S309.^163^ It has been suggested that camel‐derived nanosomes, along with bovine‐derived antibodies, can effectively reduce the chances of immune escape caused by Omicron infection.^164^

RBD mutations in Omicron variants lead to escape against some monoclonal antibodies. For example, Q493R could lead to immune escape of Omicron from LY‐CoV555/CT‐P59, Q493R/S477N results in the immune escape from LY‐CoV16/ COV2‐2196/REGN10933, G339D contributes to the immune escape from S309, G446S gives rise to the immune evasion from COV2‐2130/ REGN10987, and so on.^165^ The failure of the Omicron variant recognized by many NTD and RBD antibodies is probably related to its stable structure, local conformation, hydrophobic microenvironment, and so on.^52^ Single mutations analysis of the Omicron variant suggests that Bamlanivimab/Etesevimab is ineffective against the Omicron variants. Meanwhile, various US FDA‐approved monoclonal antibodies are ineffective against the Omicron variant, including REGN10933, REGN10987, LY‐CoV555, LY‐CoV016, and CT‐P59.^154^ A related study confirmed that approximately 85% of 247 human RBD mAbs failed to bind with the Omicron variant.^166^

Omicron RBD has a solid binding capacity to ACE2.^167^ The neutralization of the Omicron variant by antibodies from vaccination was evaluated with VSV (pseudotyped recombinant vesicular stomatitis virus) based pseudovirus.^168^ Monoclonal antibodies are effective against Omicron variants, although they are expensive and unevenly distributed globally.^17^

#### 5.2.2 Omicron variants along with the protective effect of vaccines

Cancer patients and children are immunocompromised and susceptible to Omicron infection. Currently, vaccinations, especially the booster vaccination, offer broad protection against Omicron variants. Mohiuddin and Kasahara^115^ showed that Omicron variants accelerate cellular senescence in cancer patients, promote oxidative stress, and aggravate patient complications. Protective antibodies produced after mRNA‐1273 vaccination in patients with multiple myeloma developed severe breakthrough infections 10 weeks after vaccination.^169^ After two doses of mRNA vaccination to Non‐small cell lung cancer (NSCLC) patients and healthy counterparts, NSCLC patients had lower live virus‐neutralizing activity than healthy counterparts.^129^ Cancer patients infected with the Omicron variant showed strong nAb resistance after two doses of the mRNA vaccination. In contrast, booster vaccination increased nAb titers in cancer patients, which provided broad protection against cancer patients.^92^

A related study in South Africa showed that children infected with the Omicron variant had higher hospitalization rates than those infected with other VOCs.^29^ In Berlin, the number of children infected with the Omicron variant drastically increased.^27^ It is possible that mutations, low vaccination rates, previous waves of VOCs infection, immunodeficiency, and other factors have led to increased hospitalization rates in children infected with Omicron variants^28^, ^170^ In the United States, the severity of infection in children (under 5 years old) were significantly lower during the Omicron variant wave than during the Delta variant wave.^26^ For children infected with the Omicron variant, the number of children's visits to the emergency department and hospitalization rates (under 5 years old) were also significantly lower than for those infected with the Delta variant.^31^ One study showed different responses in children (6–11 years old) vaccinated with varying doses of mRNA vaccines. Cross‐VOC antibody responses were observed at 50 and 100 μg vaccination, and humoral immunity to Omicron was observed at 100 μg vaccination.^171^ Convalescent children who have been infected with SARS‐CoV‐2 and children vaccinated with two doses of BNT162b2 have reduced sensitivity to serum antibodies against the Omicron variants and have been more vulnerable to breakthrough infection.^131^

A retrospective study conducted live virus and pseudovirus neutralization tests on the Omicron variant showed that the neutralizing effect of Omicron (B.1.1.529) variant decreased in serum from people who were vaccinated with BNT162b2 vaccine, mRNA‐1273 vaccine, Sinopharm BBIBP‐CorV vaccine, and the neutralizing activity against Omicron increased after booster vaccination.^87^ Vaccination with the third dose of mRNA produced a strong cross‐neutralizing effect on the Omicron variant,^78^, ^80^, ^95^ significantly increased serum antibody titers and reduced the escape of neutralizing antibodies from the Omicron variant.^98^, ^108^, ^172^ Vaccination with three doses of the mRNA vaccine BNT162b2 effectively increased neutralizing antibody levels against Omicron.^81^ Vaccination with two doses of BNT162b2 (>5 months) showed no neutralizing activity against the Omicron variant. After vaccination with booster vaccine, the titer of neutralizing antibody against Omicron variant was 100 times higher than that of the second dose.^74^ Moreover, after two doses of mRNA‐1273 vaccination followed by an mRNA‐1273 booster immunization, a significant increase in neutralizing antibody activity against the Omicron variant was observed, just fivefold lower than that of D614G.^173^ After two doses of BNT162b2 vaccination, antibody neutralizing titers in serum against the Omicron variant were reduced more than 22‐fold compared with that of WT, whereas a third booster dose was also effective in preventing the Omicron variant.^81^ Vaccination with the BBIBP‐CorV booster increased the neutralizing activity of the Omicron variant by approximately 3.3‐fold.^88^ After two doses of mRNA vaccine, the neutralizing activity of antibodies against the Omicron variant was absent or significantly decreased in vaccinees, who then received a booster dose, and neutralizing antibody activity against Omicron was greatly enhanced.^105^

Neutralizing activity against the Omicron variant was detected in convalescent serum.^84^ Omicron variants produce an escape response to neutralizing antibodies initiated by BNT162b2 or CoronaVac.^77^ One study has compared sera from COVID‐19 patients one year after the original D614G strain infection, where the former showed lower neutralizing activity against the Omicron variant than the latter.^100^ Chen et al.^131^ conducted a live virus neutralization test of serum from children who recovered from SARS‐CoV‐2 infection and children who received two doses of the BNT162b2 vaccine. The results showed that serum antibodies reached the detectable threshold in only 26.7% of the recovered children with SARS‐CoV‐2 infection and 38.2% of children who had received two doses of BNT162b2 vaccine. These results showed that neutralizing antibody activity against Omicron of sera from children who recovered from COVID‐19 and received two doses of BNT162b2 vaccine had remarkably decreased.^131^

All the above studies suggest that vaccination, especially booster vaccination, enhances neutralizing antibody activity against Omicron variants. Infection elicits T cell responses that cross‐recognize Omicron variants^127^ and multiple spike‐mutated VOCs also increase the breadth of antibody cross‐neutralization.^118^

Rössler et al.^82^ showed that cross‐neutralizing reactions against the Omicron variants were observed in homologous BNT162b2 vaccine recipients or heterologous ChAdOx1‐S‐BNT162b2 vaccine recipients. Heterologous vaccination with booster doses (recombinant adenovirus vector vaccine Ad26.CoV2.S, mRNA vaccine BNT162b2, recombinant adenovirus vector ChAdOx1 nCoV‐19 vaccine AZD1222, CoronaVac vaccine) induced a significant increase in antibody‐neutralizing activity compared with homologous immunization.^85^ The serum from the convalescent who received two doses of inactivated virus vaccine had poor neutralizing antibody activity against the Omicron variant. However, homologous inactivated vaccine booster or heterologous booster protein subunit vaccine (ZF2001) could significantly increase neutralizing antibody activity against the Omicron variant.^90^

## 6 FURTHER STRESS AND RISK BROUGHT BY OMICRON

*Omicron brings a more significant medical burden*: In November 2021, a rapid rise of Omicron cases happened in Gauteng, South Africa.^174^ Since Omicron was first reported by the WHO on November 24, 2021, Omicron cases have spread to more than 90 countries in a month.^175^ Omicron variants have increased transmissibility, extensive immune escape, and a potentially altered host range.^176^, ^177^ SARS‐CoV‐2 transmission dynamics in South Africa reconstructed by the model inference system indicated that the infection capacity of Omicron was 100.3% higher than the WT, 36.5% higher than Delta, and Omicron also eroded 63.7% of population immunity in Gauteng.^11^ Although the clinical symptoms caused by Omicron are mild, higher infection rates of hospital staff and more hospitalizations have overwhelmed the healthcare systems in many countries.^178^, ^179^, ^180^, ^181^, ^182^ The Omicron breakthrough infections had a significantly higher proportion than other variants in clinical cases. For example, two travelers vaccinated with BNT162b2 were detected positive for Omicron in Hong Kong,^174^ and another case is that a doctor in Israel receiving three doses of BNT162b2 was also infected with the Omicron, and he even infected another person.^183^ As of December 10, 2021, an epidemiological investigation found that 82.5% (66 out of 80) of Omicron‐positive patients had no international travel history in South Korea; therefore, the omicron case in South Korea may be more than reported.^184^ As of December 9, 2021, Denmark had confirmed 785 cases of the Omicron variant, most of which were fully vaccinated (76%) or boosted (7.1%), and 34 (4.3%) cases had previously been infected with SARS‐CoV‐2.^185^ On December 28, 2021, the number of new Omicron cases in India was 9195/day, after which the number surged to 264,202/day on January 13, 2022.^186^

*Several factors may account for the emergence of Omicron*: low vaccination rates in low‐income countries, many immunocompromised individuals, and inadequate health‐related infrastructure to cope with the worsening epidemic. At the same time, the management of booster vaccines exacerbates global vaccine inequalities, so the international health agencies must take action to provide more vaccines for countries with the lowest vaccination rates and the highest susceptibility to SARS‐CoV‐2 and its variants.^187^

*Coinfection*: There may be simultaneous infections of Omicron and Delta, resulting in a simple combinatorial variant of double‐spiked spikes called “Delmicron.” Israel reported a woman infected with both the coronavirus and the flu named “flurona.” However, these require thorough investigation before conclusions can be drawn.^188^

*Investigation of time and space*: Computer models play an important role in predicting Omicron evolution and transmission trend. The Wills‐Riley model, an airborne disease risk spatiotemporal willingness model, helped to solve the spatiotemporal problem of SARS‐CoV‐2 infection risk in ventilated indoor environments.^189^ The provincial WKDE model retrospectively analyzed the entire dynamic process of Omicron transmission in South Africa. It provided a scientific reference for controlling the SARS‐CoV‐2 variant through a simulation comparison of how to control the spread of Omicron under different scenarios with different levels of Omicron protection measures and vaccination rates.^190^

*The mental impact of Omicron on COVID‐19 patients*: Several studies have pointed out that the side effects of SARS‐CoV‐2 epidemics include worsening depression, anxiety, obsessive‐compulsive symptoms, and posttraumatic stress disorder symptoms; An increasing number of COVID‐19 patients exhibit extreme burnout and even suicide.^191^

## 7 PROSPECTS AND PERSPECTIVES

Preventive and control measures during the Omicron wave especially BA.4 and BA.5 should be more positive and scientific, measures including vaccination, increased ventilation, timely detection and isolation, wearing masks in indoor public places, washing hands, and keeping social distance from others should also be taken. Measures carried out in past influenza pandemics have provided many valuable suggestions.^192^ Vaccination, especially booster vaccination, is essential to control the spread of Omicron.^192^ Countries are called upon to unite to fight the Omicron variant and remove restrictions on the import and export of vaccines to achieve vaccine equity.^193^ In addition to primary two doses vaccination, booster vaccination, in particular heterologous booster vaccination, would mitigate the spread of the Omicron variant.^194^ Developed countries are expected to help countries with inadequate medical infrastructure, vaccine shortages, low vaccination rates, and high infection rates.^187^, ^195^

Public places such as schools, hospitals, restaurants, hotels, buses, subways, and movie theaters are high‐risk environments for VOC transmission. The CDC requires the PCR test results 24 h before arrival and 7‐day self‐isolation after arrival in the United States.^196^ Related studies have shown that N95 masks can prevent infection and alleviate Omicron transmission in hospitals after daily testing.^197^ High‐performance nanosystems (NS) have been designed for manufacturing nanoprotective equipment (masks, gloves, nanoemulsifying disinfectants, etc.) and intended to delay the infectivity of Omicron variants.^198^ It is necessary to take strict prevention and control measures for the residence and community where SARS‐CoV‐2 positive patients were found.^199^

Children and the elderly with weaker immunity are susceptible to the Omicron variant. The WHO recommends that the aged, especially those over 60 years old, should not travel during the Omicron pandemic.^196^, ^200^ It is recommended that children and seniors receive a booster vaccination as soon as possible to boost immunity by expanding the availability of childhood vaccines.^178^ Omicron is hoped to help end the pandemic,^68^ although the current situation is still unstable and challenging.^201^

## CONFLICT OF INTEREST

The authors declare that they have no conflict of interest.

## AUTHOR CONTRIBUTION

H. F., Y. T., L. S., and Y. Z. designed the research; A. X., B. H., and F. L. read and analyzed the papers; A. S., W. L., and X. A. participated in discussion; A. X., B. H., F. L., S. W., and H. F. wrote and revised the manuscript. All authors have read and approved the article. A. X., B. H., F. L., and S. W. contributed equally to this work.

## ETHICS STATEMENT

Not applicable.

## Data Availability

The data included in this study are available upon request from the corresponding author.
